# Gastrointestinal Tract As Entry Route for Hantavirus Infection

**DOI:** 10.3389/fmicb.2017.01721

**Published:** 2017-09-08

**Authors:** Peter T. Witkowski, Casey C. Perley, Rebecca L. Brocato, Jay W. Hooper, Christian Jürgensen, Jörg-Dieter Schulzke, Detlev H. Krüger, Roland Bücker

**Affiliations:** ^1^Institute of Virology, Charité – Universitätsmedizin Berlin Berlin, Germany; ^2^Virology Division, United States Army Medical Research Institute of Infectious Diseases Fort Detrick, MD, United States; ^3^Division of Hepatology and Gastroenterology, Charité – Universitätsmedizin Berlin Berlin, Germany; ^4^Institute of Clinical Physiology, Charité – Universitätsmedizin Berlin Berlin, Germany

**Keywords:** hantavirus, puumala virus, hemorrhagic fever with renal syndrome, virus entry, gastrointestinal infection, gastric barrier, tight junction, hamster model

## Abstract

**Background:** Hantaviruses are zoonotic agents that cause hemorrhagic fevers and are thought to be transmitted to humans by exposure to aerosolized excreta of infected rodents. Puumala virus (PUUV) is the predominant endemic hantavirus in Europe. A large proportion of PUUV-infected patients suffer from gastrointestinal symptoms of unclear origin. In this study we demonstrate that PUUV infection can occur via the alimentary tract.

**Methods:** We investigated susceptibility of the human small intestinal epithelium for PUUV infection and analyzed the resistance of virions to gastric juice. As model for intestinal virus translocation we performed infection experiments with human intestinal Caco-2 monolayers. In animal experiments we infected Syrian hamsters with PUUV via the intragastric route and tested seroconversion and protective immunity against subsequent Andes virus challenge.

**Results:** PUUV retained infectivity in gastric juice at pH >3. The virus invaded Caco-2 monolayers in association with endosomal antigen EEA1, followed by virus replication and loss of epithelial barrier function with basolateral virus occurrence. Cellular disturbance and depletion of the tight junction protein ZO-1 appeared after prolonged infection, leading to paracellular leakage (leak flux diarrhea). Moreover, animal experiments led to dose-dependent seroconversion and protection against lethal Andes virus challenge.

**Conclusions:** We provide evidence that hantavirus can infect the organism via the alimentary tract and suggest a novel aspect of hantavirus infection and pathogenesis.

**Significance:** Hantaviruses are zoonotic pathogens causing severe hemorrhagic fevers worldwide. They are transmitted to humans by small mammals. To date, these viruses were thought to infect exclusively through the airborne route by inhalation of aerosols from infectious animal droppings or by rodent bites. In our work we could show that the alimentary tract is an alternative path of infection for hantaviruses, meaning a new association of virus and disease. These findings have impact on current textbook knowledge and bring many implications for hantavirus epidemiology and outbreak prevention measures.

## Introduction

Hantaviruses are zoonotic viruses harbored by small mammals such as, rodents, shrews, or bats. They can cause disease in humans, leading to hemorrhagic fevers of varied severity. Old World hantavirus infections usually lead to Hemorrhagic Fever with Renal Syndrome (HFRS), with case fatality rates of up to 15%, while the clinical course of New World hantavirus infection is mainly linked with Hantavirus Cardiopulmonary Syndrome (HCPS) and case fatality rates of up to 50%. The symptomatology of both manifestations is not strict and mixed clinical courses, as well as asymptomatic infection can occur (Kruger et al., 2015).

In Europe most cases of hantavirus disease are caused by one of two hantavirus species. Human infection with Puumala virus (PUUV), which is harbored by the bank vole (*Myodes glareolus*), typically leads to less severe disease, while individuals infected with Dobrava-Belgrade virus (DOBV), whose three genotypes *Dobrava, Kurkino*, and *Sochi* are carried by rodents of the mouse genus *Apodemus*, are more likely to exhibit severe symptoms (Kruger et al., 2015). Pathogenic hantaviruses are generally thought to enter the human body by inhalation of contaminated droppings from infected host animals, followed by infection of the lung epithelium. Moreover, in rare cases, rodent bites were reported to be the cause of infection (Vaheri et al., 2013).

Hantaviruses preferentially use different β-integrins and CD55/DAF for cell entry (Gavrilovskaya et al., 1999; Krautkrämer and Zeier, 2008; Raftery et al., 2014). However, the presence of some of the receptor molecules on the basolateral side of the affected tissues requires effective disruption, or at least penetration, of the cell barrier, as shown *in vitro* for different tissue types (Krautkrämer et al., 2012). Interference with cellular barrier function of infected tissues can lead to capillary leakage and is a crucial aspect of hantavirus pathogenesis (Vaheri et al., 2013). As a prototypical member of the genus *Orthohantavirus*, Hantaan virus, was shown to enter cells by clathrin-dependent endocytosis after receptor-mediated binding to the cell surface (Jin et al., 2002).

Besides the typical organ preference of hantavirus disease, several studies have shown that the majority of patients exhibits hemorrhagic gastropathy (Nuutinen et al., 1992). Moreover, in the Syrian hamster model established for South American Andes virus (ANDV) intragastric inoculation can result in lethal disease, albeit with a higher 50% lethal dose (LD_50_) than the more effective intramuscular (i.m.), subcutaneous, intranasal (i.n.), and intraperitoneal delivery routes (Hooper et al., 2008). In rare cases, contact to contaminated food was named as a possible risk factor for human hantavirus infections (Ruo et al., 1994). Moreover, smoking was shown to be a risk factor for a PUUV infection in at least two studies (Van Loock et al., 1999; Vapalahti et al., 2010) and a possible ingestion of rodent feces or urine caused by poor hand hygiene was stated in this context (Clement et al., 2011). These findings indicate that the gut is affected in human hantavirus disease and that the infection via the alimentary tract might be a possible route of infection.

However, a gastrointestinal infection route for HFRS-causing hantaviruses has not been explicitly proposed or excluded for either humans or rodents. For this reason we conducted both *in vitro* and *in vivo* studies with PUUV, the main European hantavirus responsible for HFRS, to ascertain its viability as a route of infection. *In vitro* we investigated susceptibility of the human small intestinal epithelium for hantavirus infection, based on the polarized Caco-2 cell culture system, an established model for intestinal barrier function, and analyzed the resistance of virus particles to gastric juice. *In vivo* we demonstrated that intragastric infection of Syrian hamsters with PUUV can lead to seroconversion and protective immunity against subsequent lethal hantavirus challenge.

## Materials and methods

### Virus cultivation

For *in vitro* studies: PUUV strain Sotkamo was grown on Vero-E6 cells (ATCC CRL-1586; American Type Culture Collection, Manassas, USA) under standard cell culture conditions. Viruses were harvested by ultracentrifugation through a sucrose cushion, in order to remove residual cell culture components and titers were determined by focus titration, as previously described (Heider et al., 2001). For *in vivo* studies: PUUV strain K27 and ANDV strain Chile-9717869 were grown on Vero-E6 cells in T-150 flasks and collected from infected-monolayer supernatants. Cell debris were removed by low speed centrifugation at 1000 × g for 10 min, and virus was twice plaque purified (Hooper et al., 2001). Virus stocks were aliquoted and stored at −60°C or colder.

### Virus inactivation by gastric juice

Gastric juice was taken from adult patients who underwent gastroscopy for other diagnostic reasons and were not treated by any acid suppressing medication. According to the Declaration of Helsinki (Ethical Principles for Medical Research Involving Human Subjects) all patients gave written informed consent. The gastric juice was stored for 1–2 h in a refrigerator after its sampling. For each experiment the juice was taken from one patient. The initial pH-value (of approx. pH 1 on average) was measured by a pH-meter. The pH was attuned to different values by use of NaOH pellets. The PUUV stock was incubated with gastric juice of different pH for given time periods of 0–15 min. Subsequently pH was set to a value of 7 and culture medium was added. The treated virus solution was serially diluted and titrated on Vero-E6 cells.

### Cell culture

Caco-2 cells (ATCC HTB-37) were cultivated at 37°C with 5% CO_2_ and maintained in minimal essential medium (MEM) with 10% fetal bovine serum, and 1% L-glutamine. For immunofluorescence assays (IFA) Caco-2 cells were grown on coverslips in 24-well plates. For measurement of transepithelial electrical resistance (TER) the cells were seeded on permeable PCF filter inserts with an area of 0.6 cm^2^ and a pore size of 0.4 μm (Millipore, Germany). The cells were grown for 21 days for differentiation toward small intestinal properties. Cell medium was replaced every 2 or 3 days. TER was measured by an ohmmeter fitted with chopstick electrodes (EVOM, World Precision Instruments, USA) before infection. Confluent cell monolayers showing epithelial resistances above 500 Ω·cm^2^ were used for experiments.

### Infection experiments on human intestinal epithelial cells

Caco-2 cells on coverslips or filter inserts were infected for 1 h at a multiplicity of infection (MOI) of 0.1–1.0. Afterwards remaining inoculum was washed away and the cells were incubated in culture medium at described conditions. TER measurements in filter inserts were conducted every 24 h. Samples for microscopy were fixated with 4% methanol-free paraformaldehyde, afterwards blocked by 25 mM glycine in phosphate buffered saline (PBS). Samples for quantitative PCR (qPCR) were collected in AVL buffer (Qiagen, Germany) in case of culture medium or in RLT buffer (Qiagen) in case of cells and stored at −80°C. For virus titration from infection kinetics culture medium was stored at −80°C.

### Quantitative RT-PCR

RNA from culture supernatants and cell lysates was extracted according to manufacturer's specifications by QIAamp Viral RNA Mini Kit and RNeasy Mini Kit, respectively. Reverse transcription was performed with M-MLV reverse transcriptase (Invitrogen, Germany). Virus quantification was conducted by real-time RT-PCR, as described before (Kramski et al., 2007).

### Fluorescence microscopy

Fluorescence staining and confocal laser-scanning microscopy (CLSM) was performed as previously described (Bücker et al., 2014). The following antibodies were used: anti-ZO-1 (Zonula occludens protein-1), anti-EEA1 (early endosomal antigen 1), anti-hantaviral nucleocapsid protein (1:100), Alexa-Fluor488 goat anti-mouse or -rabbit IgG, and Alexa-Fluor594 goat anti-mouse or -rabbit IgG (1:500; Invitrogen). Cell nuclei were stained with 4′-6-diamidino-2-phenylindole dihydrochloride (DAPI, 1:1,000).

### Epithelial apoptosis

Occurrence of apoptosis was visualized by TUNEL assay (terminal deoxynucleotidyl transferase–mediated deoxyuridine triphosphate nick-end labeling, *In-situ* Cell Death Detection Kit-Fluorescein, Roche, Germany) in Caco-2 monolayers 4 days post infection as previously described (Bücker et al., 2014). Percent of apoptotic events were calculated as ratio of all cells in a low-power field (200x magnification). Six pictures per sample containing more than 1,300 cells each were counted for the analysis.

### Animal infection experiments

Female Syrian golden hamsters, age 6–8 weeks (Envigo, Indianapolis, USA) were anesthetized by inhalation of vaporized isoflurane using IMPAC 6 veterinary anesthesia machine. Once anesthetized hamsters were challenged intragastrically with 1,000 PFU PUUV, 10,000 PFU PUUV, or 10,000 PFU γ-irradiated PUUV (3 × 10^6^ rad) diluted in 1 mL sterile PBS delivered by a 3 mL syringe with a 2-inch 18 gauge gavage needle. Steps were taken to mitigate non-gastric routes of exposure (aerosol, upper respiratory tract) by using a clean needle for each hamster, loading the needles prior to removing hamsters from the anesthesia chamber, and blotting the gavage needle prior to use. The ID99 for PUUV intranasal infection is a few hundred viruses (unpublished data, Hooper lab), making infection by upper respiratory tract contamination unlikely with these precautions. Forty-two days post PUUV infection hamsters were challenged i.m. (caudal thigh) with 200 PFU ANDV diluted in 0.2 mL sterile PBS delivered with a 1 mL syringe with a 25-gauge five-eighths-inch needle. All work involving hamsters were performed in an animal biosafety level 4 (ABSL-4) facility. Euthanasia was performed on hamsters meeting early endpoint criteria.

In order to test if the inoculation procedure can affect the pH of gastric juice we administered 1 mL of PBS intragastrically to three hamsters, and euthanized them within 10 min of administration. The stomach contents (of both the forestomach and glandular stomach) were removed and centrifuged at high speed to isolate the gastric juice. The pH of the gastric juice was then compared for the hamsters that had (*n* = 3) and had not (*n* = 3) received PBS. The pH of the forestomach was essentially unchanged (1 mL PBS intragastrically: pH 1.3 vs. untreated: pH 1.5), and the pH of the stomach was similar (1 mL PBS intragastrically: pH 5.3 vs. untreated: pH 6.0) between the two groups. These data indicate that the volume of PBS used for the intragastric inoculation did not significantly affect the acidity of either the hamster forestomach or glandular stomach.

### N-specific ELISA assay

ELISA plates (Costar, United States) were coated with recombinant PUUV N-antigen in carbonate buffer (pH 9.6) overnight at 4°C. Plates were blocked with PBS, 5% skim milk, and 0.05% Tween 20 (blocking solution) for 1 h at 37°C, washed once with PBS and 0.05% Tween 20 (wash solution), and incubated with hamster sera (beginning with a 1:100 dilution) diluted in blocking solution plus 2% *Escherichia coli* lysate for 30 min at 37°C. Plates were washed 3 times with wash buffer and incubated for 30 min at 37°C with horseradish peroxidase-conjugated goat anti-hamster IgG [Kikegaard & Perry Laboratories (KPL), USA] in blocking solution. Plates were washed 3 times with wash buffer and incubated for 10 min at room temperature with tetra-methylbenzidine substrate (KPL). The colorimetric reaction was stopped by adding Stop solution (KPL) and the absorbance at 450 nm was determined. The specific sum optical density (OD) was calculated by adding the background subtracted OD values, for dilutions whose OD was greater than the mean OD for serum samples from negative-control wells plus 3 standard deviations. The PUUV N-antigen was used to detect not only PUUV but also ANDV N-specific antibody responses as previously reported (Hooper et al., 2001). Hamster sera were heat inactivated before testing by ELISA.

### Statistics

Data are expressed as mean values ± standard error of the mean. Statistical analysis was performed using 2-tailed Student *t*-test. *P* ≤ 0.05 was considered to be statistically significant. GraphPad Prism 6 software was used for the analysis.

### Ethics statements

#### Animal subjects

This study was carried out in accordance with the recommendations of Guide for the Care and Use of Laboratory Animals, National Research Council, 2011. The procedures used in this animal research were approved by the local animal welfare body known as the USAMRIID Institutional Animal Care and Use Committee (IACUC).

#### Human subjects

This study was carried out in accordance with the recommendations of GCP guidelines, Ethics Committee of the Charité, with written informed consent from all subjects. All subjects gave written informed consent in accordance with the Declaration of Helsinki.

## Results

### Susceptibility of hantavirus toward gastric juice

**Figure 1:**
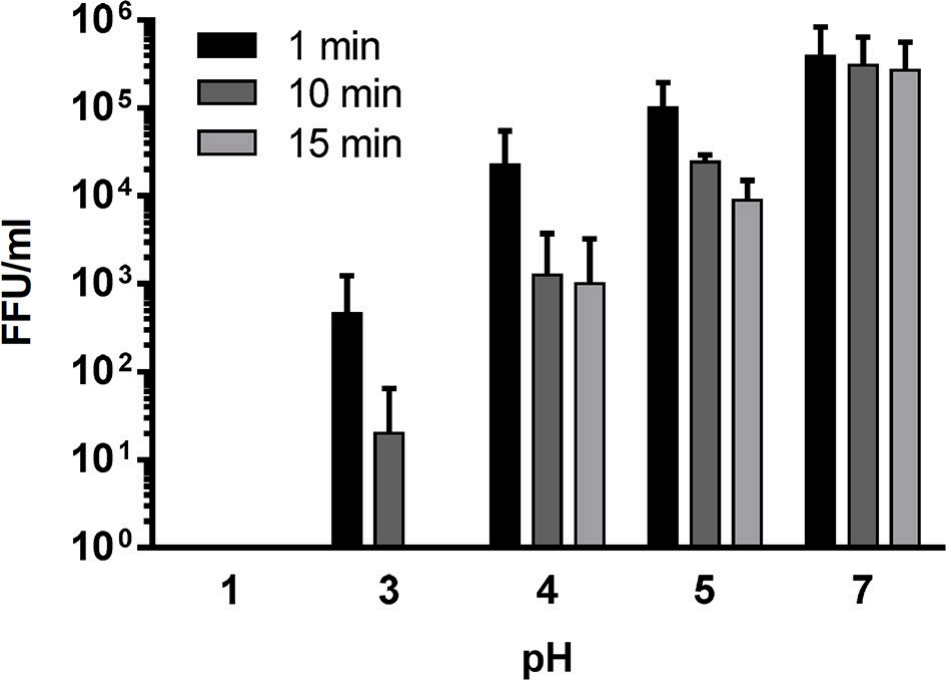
Hantavirus survival in gastric juice. Infectious dose of 10^6^ Puumala virus particles was suspended to pure gastric juice set to pH 1–7 with NaOH for 1, 10, or 15 min. After given incubation intervals the gastric juice was neutralized with NaOH and the virus suspension was used to infect VERO-E6 cells for focus titration of infectious particles (*n* = 4). As at low pH the inactivation activity was expected to be high pH 2 was omitted to save valuable sample volume.

PUUV survival in the gastric lumen was tested *in vitro* by incubation of virus stocks in human gastric juice of varying pH. The antiviral activity of gastric juice was effective at low pH between 1.0 and 3.0, with no virus surviving an exposure as short as 1 min. PUUV did survive exposure at pH values of 4.0 or 5.0 with an effective titer reduction below 1 log_10_ at pH 7.0 (Figure 1).

### Virus replication in Caco-2 monolayers

**Figure 2:**
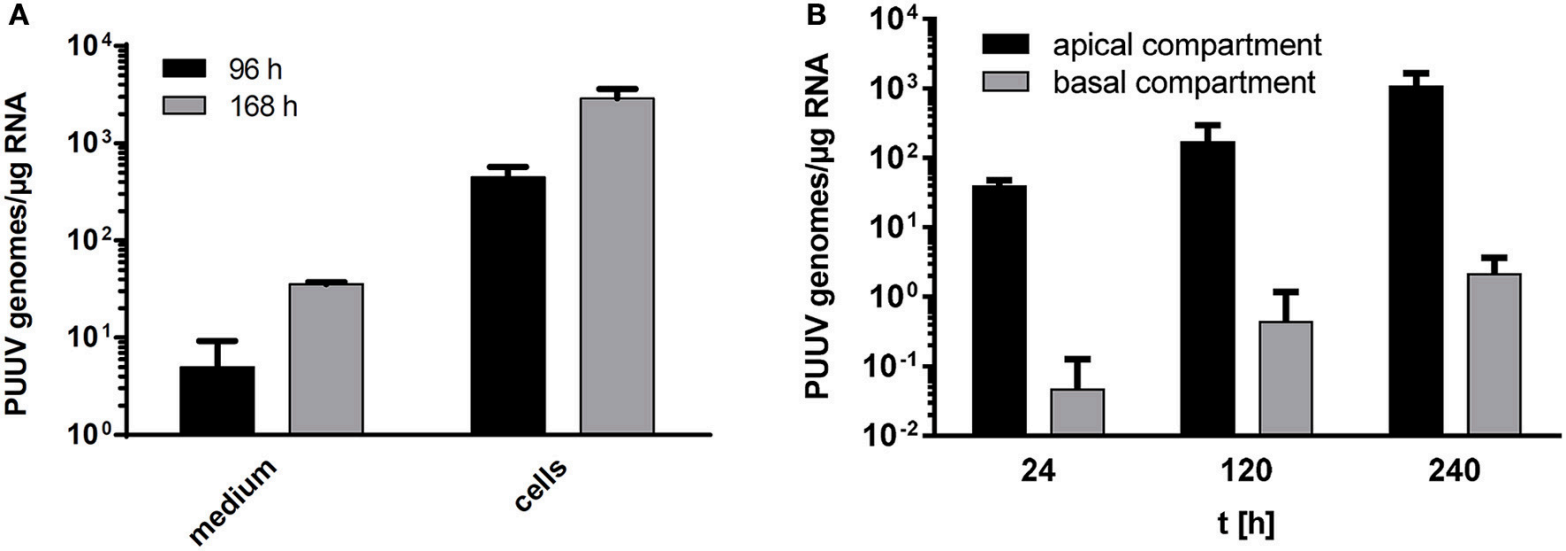
**(A)** Growth kinetics of hantaviruses in Caco-2 cells. Caco-2 cells growing on cell culture slides were infected with Puumala virus at MOI of 0.1. Viral replication was monitored by qPCR in culture medium and cells (*n* = 3). **(B)** Translocation of hantavirus through polarized Caco-2 monolayers. Caco-2 cells growing on filter inserts for at least 3 weeks were infected by Puumala virus at MOI 0.1. Apical and basal medium were collected at 24, 120, and 240 h p.i. and investigated for viral replication by qPCR (*n* = 3).

Prior to beginning experiments we confirmed that Caco-2 cells expressed ß1- and ß3-integrins as well as CD55/DAF, the receptors preferentially used by hantaviruses for entry, on their surface (data not shown). Experimental infection of human intestinal cells, Caco-2, with PUUV at MOI 0.1 revealed a time-dependent increase of intra- and extracellular viral load by quantitative RT-PCR assays (Figure 2A). Titration of supernatant confirmed the increase of viable virus progeny (data not shown). The intracellular localization of hantavirus in Caco-2 cells could also be visualized by CLSM between 24 and 96 h post infection (p.i.). Translocation of PUUV through Caco-2 monolayers after apical infection could be shown by qRT-PCR detection of viral RNA in basal culture medium. The basal release of viruses was detectable after 24 h and it increased by 2 log_10_ over the next 216 h (Figure 2B).

**Figure 3:**
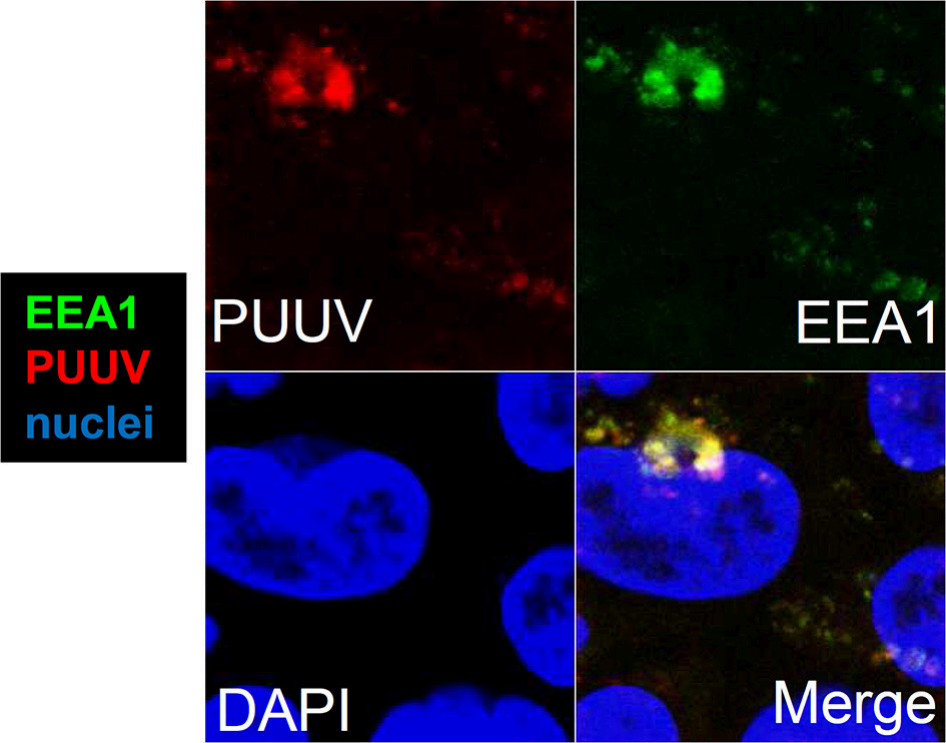
Intracellular localization of hantavirus in Caco-2 cells. Confocal laser-scanning microscopy of infected cell monolayers. Caco-2 cells growing on coverslips were infected with Puumala virus at MOI of 0.1. The cells were fixed with methanol-free formaldehyde and stained with antibodies against PUUV nucleocapsid protein (red) and early endosomal antigen 1 (EEA1, green). Cell nuclei were stained by DAPI (blue).

Hantavirus antigens were found intracellularly in co-localization with EEA1, demonstrating endosomal localization of virus particles (Figure 3). In cells infected with UV-inactivated virus such signals were not present (data not shown).

### Epithelial barrier dysfunction in infected Caco-2 monolayers

**Figure 4:**
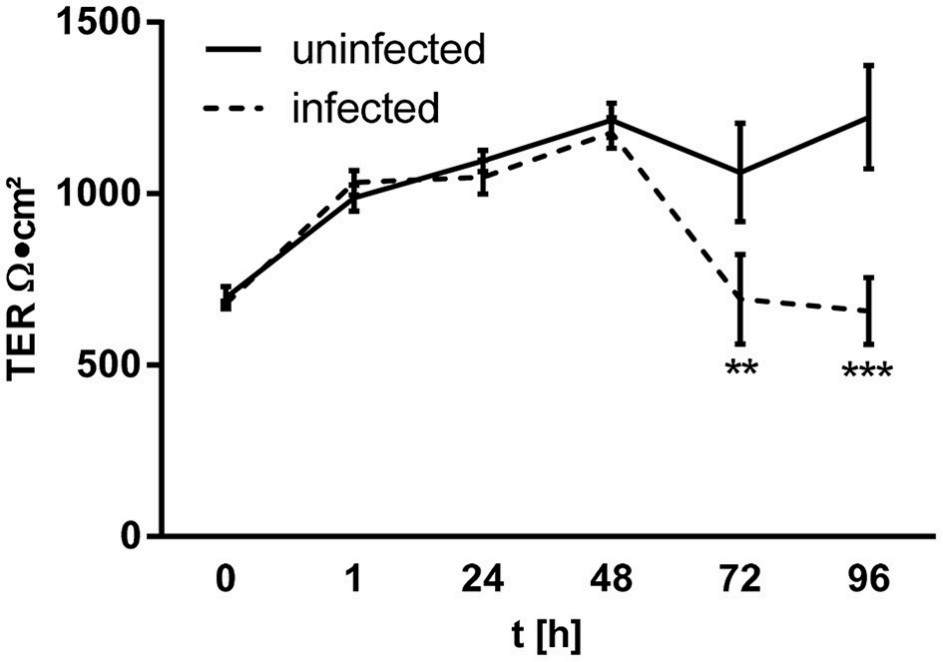
Epithelial barrier dysfunction in infected Caco-2 monolayers. Caco-2 cells growing on filter inserts were infected by Puumala virus at MOI 0.1. Transepithelial electrical resistance (TER) was measured during infection with chopstick electrodes (*n* = 4).

**Figure 5:**
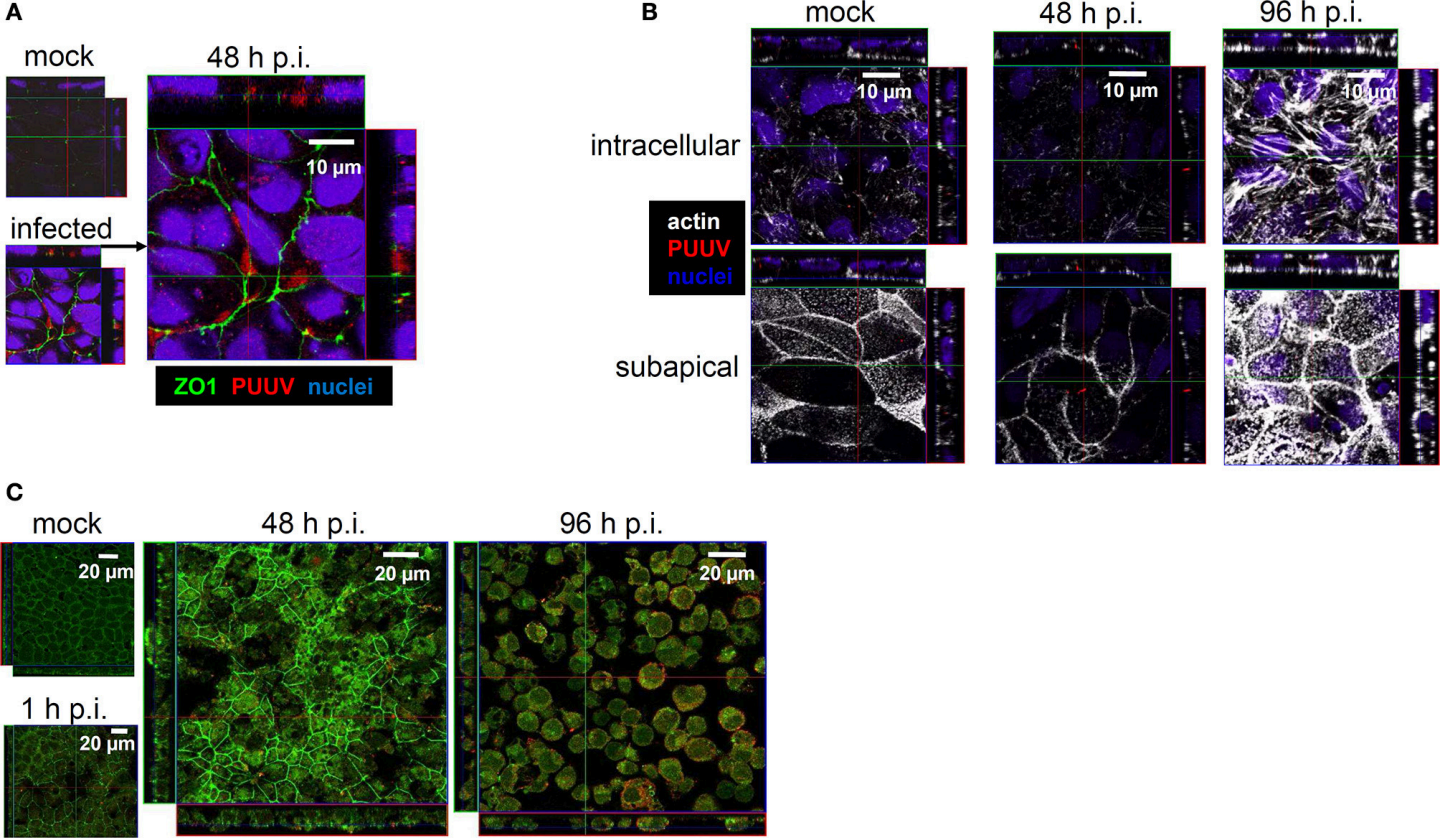
**(A)** Tight junction disturbance. Caco-2 cells grown on filter inserts were infected with Puumala virus at MOI of 0.1 and analyzed by confocal laser-scanning microscopy. At 48 h cells were fixed and stained for PUUV nucleocapsid protein (red) and zonula occludens protein 1 (green). Cell nuclei were stained by DAPI (blue). **(B)** Cytoskeleton rearrangements in infected Caco-2 monolayers. Caco-2 cells were infected with PUUV at MOI of 0.1. At different time points the cells were fixed with methanol-free formaldehyde and stained with antibodies against PUUV nucleocapsid protein (red) and actin (white). Cell nuclei were stained by DAPI (blue). **(C)** Leaks in Caco-2 cells monolayers at high MOI. Caco-2 cells grown on coverslips were infected with Puumala virus at MOI of 1.0. At different time points the cells were fixed and stained with antibodies against Puumala virus nucleocapsid protein (red) and zonula occludens protein 1 (green) and analyzed by confocal laser-scanning microscopy. Mock control is shown at 96 h.

Infection of Caco-2 monolayers at MOI 0.1 revealed stable TER values up to 48 h p.i. However, after 72 h p.i. the TER decreased to 60% of the initial value (Figure 4). Using CLSM, viral antigen was observed intracellularly, in close proximity to the tight junctions, which exhibit condensed zonula occludens protein-1 (ZO-1) (Figure 5A). However, clear co-localization of PUUV N-protein and ZO-1 did not occur, as evidenced by a lack of yellow signal, excluding a direct interaction. Moreover, the condensed ZO-1 staining pattern could indicate a disturbed gate function, by actomoysin-constriction-mediated tight junction protein re-distribution, which could also lead to a loss of cell-cell contacts. In CLSM analysis, cytoskeletal rearrangements were visible in response to virus infection after 48 h (Figure 5B). Cytoskelatal F-actin signal density increased during the course of the infection. After 96 h p.i. condensation of actin appeared as sign of apical membrane ruffling or actomyosin constriction of the perijunctional cytoskeleton. Close-by the basolateral membrane the intracellular actin formed stress fibers. To exclude the possible induction of cell death by apoptosis Caco-2 monolayers were stained by TUNEL 240 h p.i. at low MOI. Apoptotic events counted in PUUV-infected monolayers did not differ significantly from mock-infected monolayers (0.46% ± 0.10% vs. 0.42% ± 0.05%, *n* = 5, *P* = 0.73).

Infection of Caco-2 monolayers with higher concentrations of PUUV (MOI 1.0) revealed earlier and more pronounced effects (Figure 5C). Here, the condensation of ZO-1 was found by 48 h p.i., followed by disappearance of ZO-1 signals, and finally cell detachment and/or cell exfoliation leading by 96 h p.i. to epithelial leakage.

### Administration of PUUV intragastrically leads to productive infection in syrian hamsters

**Figure 6:**
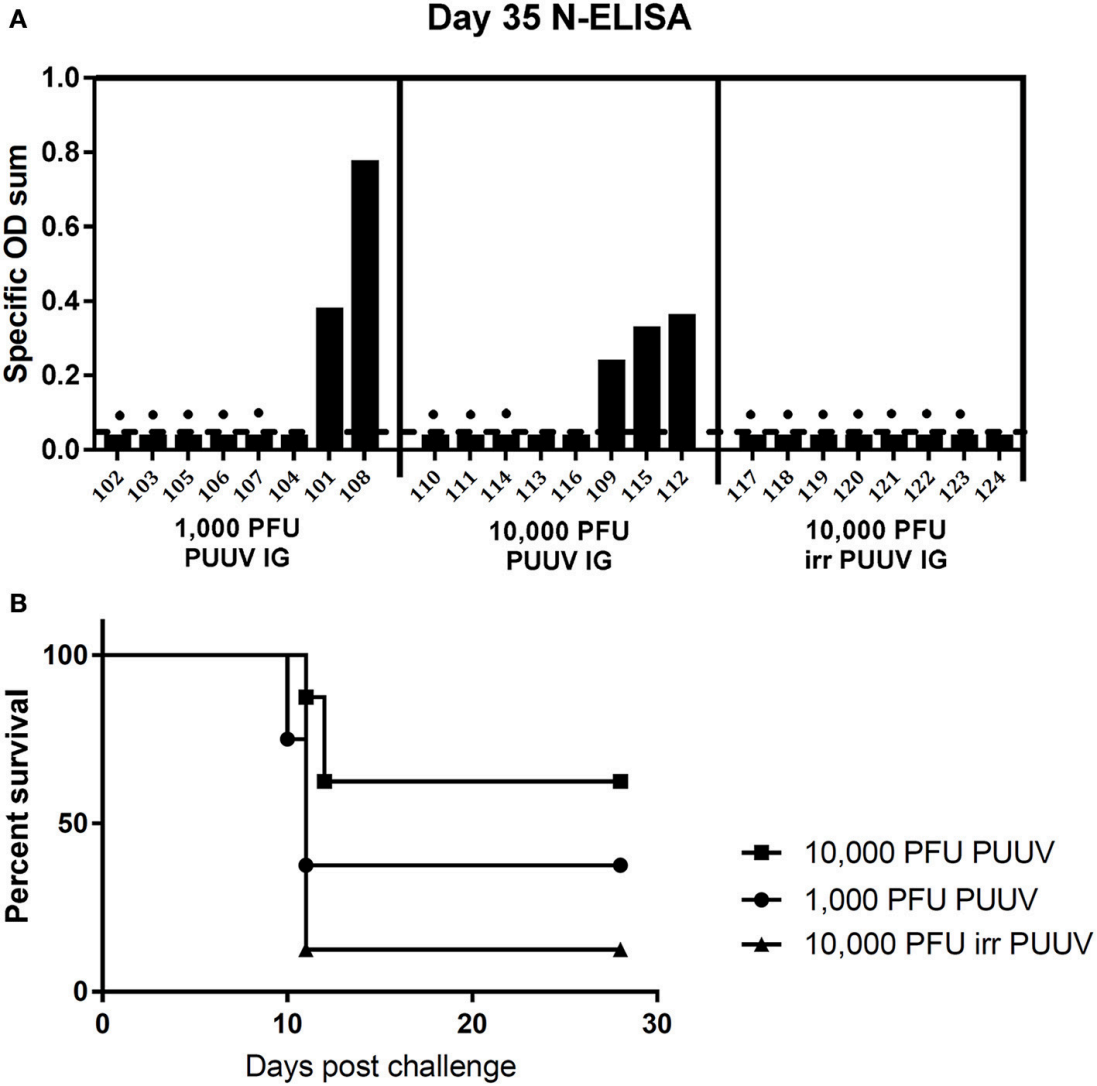
**(A)** Intragastral infection of hamster by Puumala virus. Syrian hamsters (animal identification numbers given on the x-axis) were infected with either 1,000 plaque forming units (PFU), 10,000 PFU or 10,000 PFU γ-irradiated PUUV. Thirty five days post infection hamsters were bled to test for seroconversion by N-ELISA. Dots represent hamsters for which subsequent Andes virus challenge was lethal. Specific OD sum is the sum of the optical densities (ODs) greater than background, and represents the area under the titer curve. **(B)** Survival curves for hamsters “vaccinated” intragastrically with Puumala virus. Forty two days post intragastric Puumala virus challenge, the same hamsters were challenged with 200 PFU ANDV i.m.

Groups of eight hamsters were instilled intragastrically with either 1,000 PFU PUUV or 10,000 PFU PUUV. To confirm that seroconversion was caused by active viral replication, and not input virus, a control group of eight hamsters was injected with 10,000 PFU γ-irradiated PUUV. 35 days post challenge the hamsters were bled, and seroconversion evaluated by N-ELISA (Figure 6A). 2/8 hamsters in the 1,000 PFU challenge group, and 3/8 hamsters in the 10,000 PFU challenge group seroconverted. All seroconverted hamsters had a titer of 3 log_10_. None of the hamsters instilled with irradiated virus seroconverted, confirming that viral replication occurred in seroconverted animals.

Prior infection with PUUV is able to protect animals from lethal ANDV challenge, and is a more sensitive measure of prior infection than seroconversion. To confirm that hamsters, which had seroconverted, were truly infected, at 42 days post PUUV administration all hamsters were challenged with 200 PFU of ANDV. 5/5 (100%) of the seroconverted hamsters survived ANDV challenge, confirming infection. Additional three hamsters that had been challenged with live PUUV virus survived ANDV challenge, while only one hamster, who had received irradiated virus intragastrically, survived. The survival curves for PUUV intragastrically “vaccinated” hamsters are given in Figure 6B.

## Discussion

### Stomach physiology, survival in the gastric lumen

In the human alimentary tract, entering viruses encounter gastric acid and proteolytic enzymes with gastric contents falling below pH 4 for 70% of the time (Mitchell et al., 2001). pH values in human stomach can postprandially easily increase above pH 5.0, even up to pH 7.0 in young children after consumption of food with high buffer capacity such as, milk or milk products (Mitchell et al., 2001; Bücker et al., 2012). Stomach transit time can vary between 5 min and 2 h, depending on food and liquid intake (Worsøe et al., 2011). Dyspepsia, which is diagnosed in up to 25% of western population, is treated with proton pump inhibitors (PPIs), leading to elevated pH and higher susceptibility to gastrointestinal infections (Cook, 1994).

In our study, PUUV was shown to be able to survive human gastric juice for at least 15 min at pH 4.0, though viral titers were reduced by 3 log_10_. Exposed to gastric juice at pH 5.0 for 15 min, the reduction of virus titer amounted to 2 log_10_, and at pH 7.0 less than a 1 log_10_ loss of viability was observed, suggesting the virus can survive long enough to be viably released into the duodenum. Postprandial decrease in gastric pH can lead to reduced activity of gastric proteolytic enzymes, like pepsin A or pepsin C, making the environment even more conducive to viral survival. Under postprandial or achlorhydric conditions the antimicrobial gastric barrier is vulnerable and can be overcome by pathogens (Mitchell et al., 2001; Haastrup et al., 2014). Finally, stomach conditions are dependent on the host's age, health status (gastric secretion rate), and nutritional preferences (buffer capacity of the food) leading to variable risk factors for hantavirus survival and therefore infection through the gastrointestinal tract.

In our hamster infection model, the pH of gastric juice can range between pH 1.3 and pH 6.0, the viruses have to overcome these stomach conditions after oral uptake, and also after experimental intragastric inoculation. Since the gastric pH was unchanged after inoculation (see Methods), the inoculation procedure did not contribute to a more favorable environment in the stomach lumen for virus survival. Intragastric inoculation followed by a systemic infection has been previously demonstrated in the hamster model of hantavirus pulmonary syndrome using Andes virus (Hooper et al., 2008). In hamsters the intragastric 50% lethal dose (LD_50_) of 225 plaque forming units (pfu) is higher than the LD_50_ for both intranasal (LD_50_ = 95 pfu) and intramuscular (LD_50_ = 8 pfu) infection, indicating that the intragastric route was the least efficient of the three (Hooper et al., 2008). Based on our data it is likely that gastrointestinal route of infection of animals is one possible way of virus infection also in nature. Host animals can shed virus in saliva, urine and feces (Hardestam et al., 2008). Even if studies about inter-animal transmission were performed and showed a significant male predominance among infected rodents as well as the importance of wounds and scarring, the role of ingestion could so far not be ruled out as it was never an aspect of such studies, nor could it efficiently be monitored (Calisher et al., 2007).

### Endocytotic cell entry

The small intestinal tissue, with its major absorptive function, is an entry site for pathogens including Norovirus or *Yersinia enterocolitica*. The MALT (mucosa-associated lymphoid tissue) is the site for replication and dissemination for several pathogens including HIV (Epple et al., 2010). In our experiments, Caco-2-derived human intestinal epithelial cells are capable of being infected by PUUV. The virus localized to the endocytic pathway, as visualized by co-localization of virus N-protein with EEA1, as already shown for Hantaan virus in Vero-E6 cells (Jin et al., 2002), while the membrane ruffling visible at later time points of infection could be an additional sign of high endocytic activity. Moreover, interactions of pathogenic viruses with tight junction proteins containing PDZ domains (PSD-95/Dlg/ZO-1) or CAR (Coxsackie virus and adenovirus-receptor) have been frequently described (reviewed in Zihni et al., 2014) as have interactions between old world hantaviruses and DAF/CD55 (Krautkrämer and Zeier, 2008). The data presented here, including the proximity of viral nucleocapsid protein to tight junctions together with ZO-1 ruffling, reinforce the idea that PUUV uses similar mechanisms during its infection of intestinal epithelial cells.

### Epithelial barrier dysfunction

As the result of hantavirus infection, transcytotic viral uptake occurs as seen in our cell culture infection model, even at low MOIs. In cell culture experiments using higher viral concentrations, cell detachment, and severe epithelial damage takes place with preceding cytoskeletal rearrangements and tight junction impairment. Finally, cell rounding and detachment lead to significant barrier dysfunction. Loss of cells due to apoptosis could be excluded, but cell exfoliation or shedding, as well as other cell death mechanisms like autophagy or necrosis, could play a role in the pathogenic action of hantaviruses in the gut. The latter condition may more accurately resemble clinical cases of hemorrhagic fever, where a manifestation of hantavirus disease can compromise endothelial barrier function leading to petechiae or even hemorrhages (Vaheri et al., 2013). Since disturbance of epithelial barrier function in the intestine leads to paracellular antigen influx (leaky gut concept Bücker et al., 2014), entry of further virus particles and luminal bacterial antigens is possible. This could provoke immune activation and gastrointestinal symptoms like diarrhea, vomiting, or abdominal pain, which are often found in clinical HFRS cases.

### Animal model of PUUV infection

In the Syrian hamster model of intragastric PUUV infection we were able to confirm that the virus is capable of infecting, and replicating within the animal at a challenge dose of 1,000 PFU, as measured by seroconversion and protection from lethal ANDV challenge. Given the limited number of doses tested it is not possible to determine a precise infectious dose that infects 99% (ID99) of hamsters, however, given that only 3/8 hamsters infected with 10,000 PFU PUUV survived ANDV challenge, the ID99 must be higher than that dose. This would make the ID99 for intragastric infection at least 10-fold higher than for intramuscular or intranasal (unpublished data, Hooper lab), indicating that, while intragastric infection is possible, it is less efficient than other methods used in the laboratory.

There are two main limitations of the PUUV infection of hamsters. First is that infection is transient, and it is difficult to locate live virus, viral RNA, or viral antigen in the animal post challenge. After a 1,000 PFU intranasal challenge, all animals seroconvert but live virus or viral RNA was not detected in the sera or tissues at any time-point within the first 4 weeks of infection (unpublished data Hooper lab). For this reason we are unable to confirm the hypotheses regarding PUUV disruption of the intestinal epithelial barrier during infection in an animal model. However, as none of the hamsters challenged with 10,000 PFU irradiated virus seroconverted, viral replication (though unable to be detected) must have occurred as it is a requirement for anti-PUUV antibody production. Second, PUUV infection in hamsters with viral doses close to the ID99 leads to long seroconversion times. Animals challenged with the ID99 via the i.m. or i.n. route do not seroconvert until 35 days post infection (unpublished data, Hooper lab). It is possible that the hamsters “intragastrically vaccinated” with PUUV that survived ANDV challenge, without seroconverting, had PUUV antibodies that were below our level of detection. Cross-reactivity between ANDV and PUUV antigens makes it impossible to test for the development of PUUV antibodies post ANDV challenge. It is for this reason, that survival of ANDV challenge is a more sensitive measure of productive PUUV infection.

### Hantavirus route of infection

Here, we present evidence that the oral route of infection, potentially by contaminated food, is plausible for PUUV. The results of our work imply a new aspect of hantavirus pathogenesis to be included in epidemiological considerations.

## Author contributions

Conceived and designed the experiments: DK, PW, RB, JS, and JH. Performed the experiments: PW, CP, RLB, and RB. Analyzed the data: PW, RB, CP, RLB, and JH. Important intellectual support: CJ and JH. Contributed reagents/materials/analysis tools: CJ, JS, JH, and DK. Wrote the paper: PW and RB. Critical revision and study supervision: DK and JS.

### Conflict of interest statement

The authors declare that the research was conducted in the absence of any commercial or financial relationships that could be construed as a potential conflict of interest.
